# Teaching during a pandemic: Using high‐impact writing assignments to balance rigor, engagement, flexibility, and workload

**DOI:** 10.1002/ece3.6776

**Published:** 2020-10-12

**Authors:** Julie A. Reynolds, Victor Cai, Julia Choi, Sarah Faller, Meghan Hu, Arthi Kozhumam, Jonathan Schwartzman, Ananya Vohra

**Affiliations:** ^1^ Department of Biology Duke University Durham North Carolina USA; ^2^ Program in Education Duke University Durham North Carolina USA

**Keywords:** coronavirus, COVID‐19, Eli review, peer review, science education, STEM, writing pedagogy, writing‐to‐learn

## Abstract

The COVID‐19 pandemic has created new challenges for instructors who seek high‐impact educational practices that can be facilitated online without creating excessive burdens with technology, grading, or enforcement of honor codes. These practices must also account for the possibility that some students may need to join courses asynchronously and have limited or unreliable connectivity. Of the American Association of Colleges and University's list of 11 high‐impact educational practices, writing‐intensive courses may be the easiest for science faculty to adopt during these difficult times. Not only can writing assignments promote conceptual learning, they can also deepen student engagement with the subject matter and with each other. Furthermore, writing assignments can be incredibly flexible in terms of how they are implemented online and can be designed to reduce the possibility of cheating and plagiarism. To accelerate the adoption of writing pedagogies, we summarize evidence‐based characteristics of effective writing assignments and offer a sample writing assignment from an introductory ecology course. We then suggest five strategies to help instructors manage their workload. Although the details of the sample assignment may be particular to our course, this framework is general enough to be adapted to most science courses, including those taught in‐person, those taught online, and those that must be able to switch quickly between the two.

## Graphical abstract

Writing‐intensive courses may be one of the easiest high‐impact educational practices for science faculty to adopt as they adjust to teaching during the pandemic. Writing assignments promote lasting conceptual learning, deepen student engagement, offer flexible implementation, and can be designed to reduce the possibility of cheating and plagiarism. To accelerate the adoption of writing pedagogies, we summarize characteristics of effective writing assignments, offer a sample writing assignment from an introductory ecology course, and suggest strategies to help instructors manage their workload.

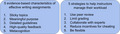

## 1 INTRODUCTION

The COVID‐19 global pandemic will require that most educators move instruction online, at least temporarily. As many of us pivot from physical to virtual classrooms, we need to focus on preserving the “high‐impact educational practices” that promote deep student engagement with their learning (Kuh, 2008). Some high‐impact practices, such as experiential education and research, are likely to be more difficult to reproduce online, whereas others, such as service‐learning projects, internships, and study abroad, may need to be postponed while we are observing physical distancing protocols. Fortunately, one high‐impact practice that can be readily facilitated online is writing, including assignments that ask students to engage with complex ideas through an iterative process of writing, feedback, and revision.

When done well, writing can promote conceptual learning, critical thinking, and communication skills (Dowd, Thompson, Schiff, & Reynolds, 2018; Reynolds, Thaiss, Katkin, & Thompson, 2012). Furthermore, writing assignments in science courses give students important opportunities to practice scientific reasoning and disciplinary ways of thinking (Dowd, Connolly, Thompson, & Reynolds, 2015; Dowd, Roy, Thompson, & Reynolds, 2015; Dowd, Thompson, & Reynolds, 2016; Reynolds & Thompson, 2011). Writing can also shift students from algorithmic learning, which is common among science students (Cracolice, Deming, & Ehlert, 2008), to more conceptual learning so that they can, for example, offer nuanced explanations rather than only definitions or calculations.

There are, understandably, barriers that instructors must overcome in order to include writing assignments as part of their teaching. Science faculty often choose not to assign writing due to concerns about the efficacy of prompts at promoting learning (Thompson et al., in review) or due to instructional constraints such as large course size, lack of expertise in teaching writing, or an overloaded curriculum (Finkenstaedt‐Quinn et al., in review). However, as new challenges arise as a result of the pandemic, instructors seek high‐impact practices that can be facilitated online without creating excessive burdens in preparing assignments, grading, or enforcing honor codes, all while being mindful of the possibility that some students may need to join courses asynchronously or have limited connectivity. Writing assignments are a viable option for science instructors to consider.

To address faculty concerns about the value of writing at promoting learning, we present five evidence‐based characteristics of effective writing assignments and illustrate these characteristics with a sample writing assignment from an introductory ecology course. We then offer instructors five strategies for managing their workload. Although the details of the sample assignment may be particular to our course, this framework is general enough to be adapted to most science courses, including those taught in‐person, online, and hybrids of the two.

## 2 CONTEXT FOR THE SAMPLE WRITING ASSIGNMENT

“*Ecology of Human Health*” is an introductory ecology course designed for undergraduates interested in understanding how human health is linked to the environments in which we live. The three major sections of the course are food security, disease ecology, and climate change, taught in that order. However, in January 2020 when news hit about a novel coronavirus that emerged in Wuhan, China, we quickly adjusted our course schedule to discuss zoonotic diseases, age structure, metapopulations, and superspreaders. By the time, the stay‐at‐home orders were issued for our state, we had been tracking the spread of the disease for 6 weeks, having asked the question on 20 January 2020: “Are we witnessing the beginning of a pandemic?” That was also the point at which the students and instructor became cocreators—and coauthors—of a writing assignment about COVID‐19 which we present below. The instructor of the course (coauthor JAR) has taught writing‐intensive courses for 18 years and has an active research program focusing on writing‐to‐learn pedagogies.

## 3 FIVE CHARACTERISTICS OF EFFECTIVE WRITING ASSIGNMENTS

Faculty are justifiably skeptical about the efficacy of writing prompts; writing assignments are not inherently beneficial and, if not carefully designed, they can create unproductive work for both instructors and students (Bangert‐Drowns, Hurley, & Wilkinson, 2004; Reynolds & Moskovitz, 2008). Assignments that are “knowledge‐telling” are likely to be less beneficial than those that are “knowledge‐transforming” (Bereiter & Scardamalia, 1987). In knowledge‐telling assignments, students write summaries or descriptions but there may be little critical thinking involved. In contrast, knowledge‐transforming assignments require students to weigh evidence, construct an argument, or critique ideas. In general, knowledge‐transforming tasks include higher‐order cognitive activities such as applying a concept to solve a novel problem, analyzing data, evaluating claims, and synthesizing multiple pieces of information to generate new understanding (Anderson, Anson, Gonyea, & Paine, 2015; Bloom, 1956; Lemons & Lemons, 2013). The benefits of writing assignments, therefore, depend on the cognitive activities that students engage in while writing (Galbraith, 2015). The best assignments obviously have clear expectations but also require students to construct their own understanding of an issue through the iterative process of writing, receiving feedback, and revising (Anderson et al., 2015). Additionally, they require students to monitor and evaluate their thinking throughout the process (Bangert‐Drowns et al., 2004). Assignments with all or most of these components are correlated with the largest student learning gains (Gere, Limlamai, Wilson, MacDougall Saylor, & Pugh, 2019).

Here are five characteristics of effective writing assignments:

1. Sticky topics

Not all topics are suitable for writing assignments as some concepts are taught more efficiently through other methods. For example, topics that have a singular correct answer (e.g., “What is the Hardy–Weinberg equilibrium?”) may be better suited for short‐answer assessments or calculations than a full, fleshed‐out writing assignment. In contrast, writing assignments are particularly appropriate when instructors want students to grapple with topics that, for example, are inherently challenging, conceptually complex, include common misconceptions, or are rooted in threshold concepts which, once understood, allow for greater competency in a subject (Loertscher, Green, Lewis, Lin, & Minderhout, 2014; Marion et al., in preparation; Meyer & Land, 2005). These so‐called “sticky” topics warrant the time and effort of a writing assignment and the interactive feedback involved with review and revision. Another consideration for appropriate topics is to acknowledge that students are more motivated and engaged when instructors connect assignments with real‐world issues that students care about (Herrington, Oliver, & Reeves, 2003), particularly students from groups that have been historically excluded from science (e.g., Williams, Papierno, Makel, & Ceci, 2004).

**Topic for our sample writing assignment**

“When COVID‐19 swept across the globe, policy makers endeavored to flatten the curve through various strategies such as requiring physical distancing, case isolation, quarantine, mandatory face coverings, and business and school closures. To investigate the effectiveness of such strategies, scientists use mathematical models which allow them to make predictions of how —and how quickly—this disease spreads. As you have learned in class, these models are based on assumptions and estimates of a select few parameters; given what you have learned, which parameter (from any of the models we have studied) do you think would be most useful to have a more accurate estimate of? Make a well‐reasoned argument for why improving our estimates of that parameter would give policy makers a better understanding of which strategies would be most effective in flattening the curve.”

Meaningful purpose

The purpose or goal of writing can range from entertainment (e.g., fiction) and expression (e.g., poetry) to information (e.g., journalism) and persuasion (e.g., editorial). In science courses, instructors need to consider the purpose of the writing they assign. Writing assignments that ask students to summarize a complicated process in their own words (e.g., “Explain climate change to a nonspecialist audience”) may certainly inform instructors about students’ understanding, but this is an example of a knowledge‐telling exercise and students may struggle to find their voice. Instructors can shift the goal of this assignment to knowledge‐transforming by asking students to construct an evidence‐based argument about whether or not current efforts to mitigate climate change are likely to be effective (Jang, 2007; Klein, 2004). The purpose of an academic argument is to create and share new knowledge; in this case, the new knowledge is the student's position as supported by the evidence that they have synthesized and evaluated. Another example of a knowledge‐transforming assignment would be to ask students to write about their beliefs and doubts regarding climate change and what evidence would change their minds. The purpose of this type of critical reflection is for students to synthesize and make meaning of their prior knowledge, experiences, biases, and opinions.

**Purpose of our writing assignment**

“The main purpose of this assignment is for you to develop a logical argument that is supported by evidence and reasoning (not simply citing claims made by others). To achieve this goal, you will need a solid understanding of ecological concepts and you must be able to apply critical thinking skills to solve a complex problem that doesn't have a singular or simple solution.”

- Detailed guidelines

Scaffolding involves providing detailed instructions for the various stages of the writing process. Effective writing prompts go well beyond assigning a topic; they include clear expectations about the purpose of the assignment (Melzer, 2014) as well as guidance about the audience, genre, and modes of assessment (Anderson et al., 2015).

Prompts should be explicit about who their *audience* is. Too often, college writing assignments have an actual audience comprised of only one person (i.e., the instructor) or an imagined audience (e.g., a national review panel for grant proposals). One challenge with these audiences is that students, who are novices, are being asked to feign expertise and communicate to experts, a scenario that may invoke impostor's syndrome or stereotype threat (Steele, 2011); these types of assignments may exacerbate students’ fears that they need to pretend to be someone who they know that they are not. Another challenge with this type of audience is that the power differential between the student and their audience may deny student agency, especially if students try to write what they are guessing the audience wants to hear in order to achieve their goal of a good grade. One of the best strategies for helping young writers to develop an authentic voice is to have them write to real audiences (Bereiter & Scardamalia, 1987), such as their classmates or members of their community.

The *genre* of writing should be unambiguous in writing prompts; instead of asking student to write a “paper,” we should ask for a persuasive essay, an editorial, an opinion pieces, or a critical reflection. By naming the genre, we give students insights into purpose, form, length, tone, and citation conventions. Assigning writings genres that are common within our academic disciplines serves the added purpose of socializing students into the conventions of our disciplines. In a review of over 200 studies focused on the efficacy of writing in STEM disciplines at the college level, Reynolds et al. (2012) identified two genres of writing assignments that were most strongly associated with improved learning. The first involves assignments that ask students to formulate a supported argument (e.g., Armstrong, Wallace, & Chang, 2008; Bradley, 2001; Kelly, Chen, & Prothero, 2000; Kelly & Takao, 2002; Lerner, 2007), which requires students to evaluate the strength of evidence and add their voice to the conversation by crafting a claim. The second involves assignment that requires critical reflections (e.g., Bangert‐Drowns et al., 2004; Lerch, Bilics, & Colley, 2006) which ask students to identify and challenge their thoughts and beliefs. Given that belief systems may mediate or moderate learning (Bransford, Brown, & Cocking, 2000), writing is a good way to let student examine these complicated interactions.

Finally, instructors should be explicit about *how the writing will be assessed*, what modes of feedback they will receive, and the timing of that feedback (Borgman & McArdle, 2020).

**Guidelines for our writing assignment**

“Write a 500‐word editorial for a newspaper.

Tips:

A complete answer does not have to involve any mathematics but, if included, must be explained conceptually. The focus of your editorial should be in applying your understanding of ecological concepts to infer what the most important limitation is to our current understanding. Furthermore, there is no single answer, and the strength of your writing will be based on the argument you construct.

Cite all sources you use. You do not need to do additional research—although you may—but you must site any sources you draw upon, including all course readings.

I strongly suggests you compose your paper in a Word document, saving drafts frequently, then paste your writing into Eli Review. Keeping copies of drafts will make the revision process easier.

Due dates:

Draft 1 uploaded to Eli Review by 10 a.m. EDT on <date>

Peer review in Eli Review by 10 a.m. EDT on <date +4 days>

Draft 2 (with revision plan) uploaded to Eli Review by 10 a.m. EDT on <date +7 days>”

- High‐quality feedback

One of the most significant barriers to assigning writing, especially in large science courses, is the time commitment required by instructors to grade or provide feedback on student writing (Moon, Gere, & Shultz, 2018). To address this barrier, we suggest integrating peer review into writing assignments, a strategy that is known to promote learning for both the one giving the feedback as well as the one receiving it (Li, Liu, & Steckelberg, 2010; Lundstrom & Baker, 2009), offering both pedagogical value and time savings for instructors (Cho & MacArthur, 2010; Cho & Cho, 2010; Finkenstaedt‐Quinn, Snyder‐White, Connor, Gere, & Shultz, 2019; Halim, Finkenstaedt‐Quinn, Olsen, Gere, & Shultz, 2018). Peer review promotes learning through a number of possible pathways; there is evidence that it encourages students to evaluate assignments more carefully (Li et al., 2010) and to focus more clearly on the overall purpose of assignments (Lundstrom & Baker, 2009). Learning gains may also be the indirect effect of increases in self‐efficacy that occur when students participate in reciprocal peer review (Ruegg, 2018).

We do not advocate peer grading or peer editing; instead we support the idea that students are highly capable of giving each other meaningful, formative feedback that both allows them to rethink and rewrite. Students must be taught how to give high‐quality peer feedback (see the appendix to Reynolds & Russell, 2008 for an example of guidelines to give to peer reviewers) and they must be motivated to invest the time and effort in that process by, for example, knowing that their peer reviews will be graded (see “Limit grading” section below).

**Feedback plan for our writing assignment**

In addition to receiving guidelines for how to conduct peer review (Reynolds & Russell, 2008), the peer‐review process is scaffolded to ensure that their feedback is focused on the issues that aligned with the learning goals for the assignment. We assigned the following four components for peer review:

1. *Trait Identification*. Select all that apply to your classmate's draft:
  - Does the writer identify a single parameter from one of the models?
  - Does the writer make an argument for why knowing more about this parameter is important?
  - Is there evidence cited in support of this argument?
  - Is the evidence cited persuasive?
  - Does the writer connect an understanding about this parameter to the relevance in policy making?
  - Are the connections that the writer makes convincing?
  - Was the writing appropriate for the target audience?
  - Were the citations complete and professionally reported?
2. *Rating Scales*: How strong is this first draft? 1–2 stars mean it needs a lot of work; 3–4 stars mean it is on target but could be enhanced with some additional attention; 5 stars mean that you think the essay would get full marks as is (this will be rare for first drafts!)
3. *Contextual Comments*: Using the “trait identification” section as a guide, give your classmates feedback on the areas in which they could make improvements to their writing. The best peer reviews offer sufficient quantity—and quality—of feedback such that your classmate knows what is missing or unclear and can make substantial improvements in their revision. TIP: The class average is to make 4 written comments per review (>200 words total) with the best reviewers offering > 5 comments.
4. *Final Comment*: If your classmate only had time for one change, which change do you think would most significantly improve the strength of their argument?

- Metacognition

Even the most carefully constructed writing assignments will not promote deep learning if students approach them with a mindset focused simply on reporting what they think they know. Students who are not practiced at self‐reflection will approach complex issues with increasingly complex—although nonetheless rote—solutions (Lemons, Reynolds, Curtin, & Bissell, 2013; Tsai, 2001). Therefore, the writing assignments that are likely to be of greatest benefit are those that explicitly promote metacognition (Bangert‐Drowns et al., 2004). Metacognition refers to “the knowledge, awareness, and control of one's own learning” (White, 1998) including students’ ability to predict how well they will do on a task based on what they know as well as an awareness of what they don't fully understand (Bransford et al., 2000). Some of the most effective assignments will naturally involve metacognitive processes such as planning what to write, monitoring the development of the narrative, and evaluating the clarity of one's own writing. Instructors can promote these practices by asking students to write brief reflections at various stages in the writing process. Peer review can also be a powerful tool to promote metacognition, particularly self‐regulated learning (Bransford et al., 2000): through the process of analyzing classmates’ writing in response to a rubric, students are better able to predict their own performance through monitoring their understanding of both the content and the expectations.

**How we promoted metacognition with our writing assignment**

We required a “Revision Plan” (to be submitted with the final draft) which has the following two components (adopted from https://elireview.com/learn/tutorials/students/using‐feedback/):

“Rate all the comments you received, on a scale of 1–5, for helpfulness. Rating your feedback serves several purposes, including deciding what feedback to use, giving feedback to reviewers regarding the helpfulness of their comments, and informing your instructor about whether or not you received useful feedback. Add the helpful comments to your revision plan.

A revision plan is simply a brief paragraph in which you reflect on the feedback you received and explain how you will use it in your revision. In other words, what did you learn through the peer‐review process that will help you make meaningful revisions? As a reminder, you are not obligated to use any of the feedback you received and considerable benefit may be derived from reading and reviewing your classmates’ writing.”

## 4 FIVE STRATEGIES FOR MANAGING THE WORKLOAD

Writing assignments, when done well, are “high‐impact pedagogical practices,” but in order for instructors to assign writing in their courses, they must address barriers to implementation such as large course size, lack of expertise in teaching writing, or overloaded curriculum. They must also address emergent challenges such as reducing opportunities for cheating online and increasing access for remote and asynchronous students. We offer the following suggestions for implementing writing assignments that can be effective even in large classes delivered online, synchronously or asynchronously. We begin with a caveat, however; with limited time, instructors must still make the trade‐off between how much content they can deliver versus how much time and effort they allocate for grappling with each topic. It is well beyond the scope of this manuscript to argue that point. Instead, we address the remaining barriers and suggest strategies for managing the workload associated with implementing writing assignments effectively and efficiently.

1. Use peer review

Peer review is pedagogically valuable regardless of course size, but in large classes, it has the added benefit of tempering instructors’ workload. There are many technologies available to facilitate the exchange of drafts for peer review, including simply assigning peer‐review groups and asking students to exchange drafts via email, shared documents, or discussion boards. We used the software Eli Review (elireview.com) as this program manages all the deadlines, can be integrated into course‐management software, such as Sakai, and offers a seamless interface for our students to upload drafts, review each other's writing, respond to feedback, create revision plans (including rating the helpfulness of their classmates’ feedback), and resubmit. More importantly, the software is designed to promote best practices in writing pedagogy, providing built‐in support for faculty. There is a tremendous amount of flexibility in how to set up the peer‐review process in Eli Review; instructors can decide whether or not to make reviews anonymous, how many peers are within a reviewing group, if late submissions are accepted and, if so, how those students are assigned to groups. The software also offers instructors with plentiful analytics, such as the number of comments reviewers make and how long those comments are, both of which could be used as proxies for student engagement. Although we have no direct experience with other peer‐review software (such as iPeer and peerScholar), these tools may be more readily available on some campuses.

- Limit grading

Another strategy for limiting instructor workload is to be very disciplined about what and how to grade. Most science faculty are under no obligation to teach writing skills, and therefore even if they assign writing, they are not obligated to grade the quality of the writing per se. As with all good teaching, assessments must align with learning outcomes. Therefore, if the primary learning outcome of a writing assignment is to assess students’ ability to use scientific evidence in support of claims, for example, then instructors can design grading rubrics to focus on those issues and not on the mechanics of writing.

Furthermore, if the goal is to encourage students to grapple with complicated scientific issues, it is reasonable to treat the writing assignment as a formative assessment and assign a grade based on how deeply students engaged with the process (e.g., meeting deadlines, writing substantive and helpful comments in peer reviews, making meaningful revisions). Some instructors may want to link writing assignments with summative assessments (via online quizzes or tests, for example) to assess content knowledge (Marion et al., in preparation).

Alternatively, instructors can provide valuable but limited feedback to students on their writing in a number of ways. One option, within Eli Review, is to endorse or contradict peer‐review comments for additional formative feedback students can use toward revision. Another option is for instructors to grade final drafts using rubrics that limit the number of factors that instructors need to attend to (~5 items is a reasonable target). To avoid time creep, instructors should resist editing student writing.

- Collaborate with experts

Another common barrier to implementing writing that science faculty cite is lack of familiarity with writing pedagogy (Finkenstaedt‐Quinn et al., in review). For those lucky enough to work at colleges and universities that have Writing Centers or Centers for Teaching and Learning, we encourage you to collaborate with these experts in crafting assignments and rubrics for your courses. These colleagues can help identify which peer‐review and plagiarism‐prevention software they support, as well as help you set up online assessments of writing using software such as Crowdmark and Gradescope. At the very least, it is useful to have a colleague who is well‐versed in writing pedagogies to review assignments and point out potential ambiguities that may distract students or make grading more problematic. For campuses that do not have these resources, we suggest the following online resources: Calibrated Peer Review's library of writing assignment (http://cpr.molsci.ucla.edu/Home, although we caution against adopting assignments without modifying them to meet your specific institutional context, see Reynolds & Moskovitz, 2008), Eli Review's learning resources (https://elireview.com/learn/), Science Writing Heuristic (https://education.uiowa.edu/science‐writing‐heuristic‐swh), and the WAC Clearinghouse (https://wac.colostate.edu/).

- Reduce incentives for cheating

An additional challenge to teaching online is to create assessments that do not need to be proctored. Writing assignments fit this bill with the added benefit that well‐crafted assignments reduce the likelihood of cheating and plagiarism. Unlike knowledge‐telling assignments or assignments with a singular correct answer that could be copied from a source, knowledge‐transforming assignments require students to stake out a position and are therefore less amenable to copy‐and‐paste answers. We suggest further reducing incidents of plagiarism by teaching students the conventions of citation in your discipline; these are often different from those in the humanities which may be the only place students have learned about disciplinary‐specific citation. To avoid reinventing the wheel, we suggest either inviting campus librarians to give short tutorials (live or recorded) or to link to existing tutorials on campus library webpages. Additionally, we suggest informing students in advance that all their writing will be run through plagiarism detection software such as iThenticate or Turnitin. Faculty who use Eli Review can download all final drafts for an assignment into a single file which they can then easily upload to iThenticate for review. We suggest doing this early in the semester and using any problems detected as a teaching moment for the entire class; we have found that this approach virtually eliminates subsequent issues with plagiarism.

- Be flexible

Finally, in these challenging times, both students and faculty will benefit from increased flexibility. Unlike proctored examinations, for example, writing assignments are inherently flexible in terms of when students can work on the assignment. We encourage instructors to spread out the deadlines for the various elements of the assignment to avoid unnecessary pressure. Under normal conditions, we have found that for short assignments (~500 words) one week is generally the minimum gap between the due dates for the first draft and the final draft, allowing several days for thoughtful peer reviews and the rest of the week for meaningful revisions. Given the unpredictable nature of teaching during a global health crisis, instructors may want to stretch out those deadlines to avoid some of the challenges we will undoubtedly face as a result of illness or unpredictable connectivity. Increasing peer‐review groups (from 2 to 3, for example) will reduce the complications if one student cannot complete their review on time.

## 5 CONCLUSION

We have claimed that of the American Association of Colleges and University's list of 11 high‐impact educational practices, the easiest for science faculty to adopt during these difficult times is writing‐intensive courses. We are not naïve in believing that it is easy to convert an existing course into one that is writing‐intensive; we acknowledge that little about teaching during a pandemic is easy. Instead, we suggest that writing assignments can be powerful tools for faculty who seek rigorous assignments that promote deep engagement with the subject matter and among students through the iterative process of writing, giving and receiving feedback, reflection, and revision. All this can be done online and asynchronously, giving students great flexibility in when they complete assignments. As for faculty, there is certainly an initial time investment involved in creating the infrastructure for writing assignments (a process that we suggest could be done in collaboration with campus writing specialists) but the workload can be managed carefully, even in large classes. The pay‐off is in providing a high‐impact experience for students even in these uncertain times.

## CONFLICT OF INTEREST

None declared.

## AUTHOR CONTRIBUTION

**Julie A. Reynolds:** Conceptualization (equal); Funding acquisition (lead); Writing‐original draft (lead); Writing‐review & editing (equal). **Victor Cai:** Conceptualization (equal); Writing‐review & editing (equal). **Julia Choi:** Conceptualization (equal); Writing‐review & editing (equal). **Sarah Faller:** Conceptualization (equal); Writing‐review & editing (equal). **Meghan Hu:** Conceptualization (equal); Writing‐review & editing (equal). **Arthi Kozhumam:** Conceptualization (equal); Writing‐review & editing (equal). **Jonathan Schwartzman:** Conceptualization (equal); Writing‐review & editing (equal). **Ananya Vohra:** Conceptualization (equal); Writing‐review & editing (equal).

## Data Availability

Not applicable.

## References

[ece36776-bib-0001] Anderson, P. , Anson, C. M. , Gonyea, R. M. , & Paine, C. (2015). The contributions of writing to learning and development: Results from a large‐scale multi‐institutional study. Research in the Teaching of English, 50(2), 199–235.

[ece36776-bib-0002] Armstrong, N. A. , Wallace, C. S. , & Chang, S. M. (2008). Learning from writing in college biology. Research in Science Education, 38, 483–499.

[ece36776-bib-0003] Bangert‐Drowns, R. L. , Hurley, M. M. , & Wilkinson, B. (2004). The effects of school‐based writing‐to‐learn interventions on academic achievement: A meta‐analysis. Review of Educational Research, 74(1), 29–58. 10.3102/00346543074001029

[ece36776-bib-0004] Bereiter, C. , & Scardamalia, M. (1987). The psychology of written composition. Hillsdale, NJ: Lawrence Erlbaum Associates.

[ece36776-bib-0005] Bloom, B. S. (1956). Taxonomy of education objectives. Boston, MA: Addison‐Wesley Longman Ltd.

[ece36776-bib-0006] Borgman, J. , & McArdle, C. (2020). Personal, accessible, responsive, strategic: Resources and strategies for online writing instructors. Boulder, CO: CSU Open Press.

[ece36776-bib-0007] Bradley, D. B. (2001). Developing research questions through grant pro‐ posal development. Educational Gerontology, 27, 569–581.

[ece36776-bib-0008] Bransford, J. D. , Brown, A. L. , & Cocking, R. R. (2000). How people learn: Brain, mind, experience, and school. Washington, DC: National Academies Press.

[ece36776-bib-0009] Cho, K. , & MacArthur, C. (2010). Student revision with peer and expert reviewing. Learning and Instruction, 20(4), 328–338. 10.1016/j.learninstruc.2009.08.006

[ece36776-bib-0010] Cho, Y. H. , & Cho, K. (2010). Peer reviewers learn from giving comments. Instructional Science, 39(5), 629–643. 10.1007/s11251-010-9146-1

[ece36776-bib-0011] Cracolice, M. S. , Deming, J. C. , & Ehlert, B. (2008). Concept learning versus problem solving: A cognitive difference. Journal of Chemical Education, 85(6), 873–878. 10.1021/ed085p873

[ece36776-bib-0012] Dowd, J. , Connolly, M. , Thompson, R. J., Jr. , & Reynolds, J. A. (2015). Improved reasoning in undergraduate writing through structured workshops. The Journal of Economics Education, 46(1), 14–27. 10.1080/00220485.2014.978924

[ece36776-bib-0013] Dowd, J. , Roy, C. , Thompson, R. J., Jr. , & Reynolds, J. A. (2015). “On course” for supporting expanded participation and improving scientific reasoning in undergraduate thesis writing. The Journal of Chemical Education, 92(1), 39–45. 10.1021/ed500298r

[ece36776-bib-0014] Dowd, J. , Thompson, R. J., Jr. , & Reynolds, J. A. (2016). Quantitative genre analysis of undergraduate theses: Uncovering different ways of writing and thinking in science disciplines. Writing across the Curriculum Journal, 27, 36–51.

[ece36776-bib-0015] Dowd, J. E. , Thompson, R. J., Jr. , Schiff, L. A. , & Reynolds, J. A. (2018). Understanding the complex relationship between critical thinking and science reasoning among undergraduate thesis writers. CBE‐Life Sciences Education, 17(1), ar4 10.1187/cbe.17-03-0052 29326103 PMC6007780

[ece36776-bib-0016] Finkenstaedt‐Quinn, S. A. , Gere, A. R. , Dowd, J. E. , Thompson, R. J. Jr. , Halim, A. S. , Reynolds, J. A. , … Shultz, G. (in review). STEM faculty views of writing in the classroom – Factors influencing use. Submitted to Science Education.

[ece36776-bib-0017] Finkenstaedt‐Quinn, S. A. , Snyder‐White, E. P. , Connor, M. C. , Gere, A. R. , & Shultz, G. V. (2019). Characterizing peer review comments and revision from a writing‐to‐learn assignment focused on Lewis structures. Journal of Chemical Education, 96(2), 227–237. 10.1021/acs.jchemed.8b00711

[ece36776-bib-0018] Galbraith, D. (2015). Conditions for writing to learn. Journal of Writing Research, 7(1), 215–266. 10.17239/jowr-2015.07.01.09

[ece36776-bib-0019] Gere, A. R. , Limlamai, N. , Wilson, E. , MacDougall Saylor, K. , & Pugh, R. (2019). Writing and conceptual learning in science: An analysis of assignments. Written Communication, 36(1), 99–135. 10.1177/0741088318804820

[ece36776-bib-0020] Halim, A. S. , Finkenstaedt‐Quinn, S. A. , Olsen, L. J. , Gere, A. R. , & Shultz, G. V. (2018). Identifying and remediating student misconceptions in introductory biology via writing‐to‐learn assignments and Peer Review. Cbe—life Sciences Education, 17(2), ar28 10.1187/cbe.17-10-0212 29749850 PMC5998326

[ece36776-bib-0021] Herrington, J. , Oliver, R. , & Reeves, T. C. (2003). Patterns of engagement in authentic online learning environments. Australian Journal of Educational Technology, 19(1), 59–71. 10.14742/ajet.1701

[ece36776-bib-0022] Jang, S. J. (2007). A study of students’ construction of science knowledge: Talk and writing in a collaborative group. Education Research, 49(1), 65–81. 10.1080/00131880701200781

[ece36776-bib-0023] Klein, P. D. (2004). Constructing scientific explanations through writing. Instructional Science, 32(3), 191–231. 10.1023/B:TRUC.0000024189.74263.bd

[ece36776-bib-0024] Kelly, G. J. , Chen, C. , & Prothero, W. (2000). The epistemological framing of a discipline: Writing science in university oceanography. Journal of Research in Science Teaching, 37, 691–718.

[ece36776-bib-0025] Kelly, G. J. , & Takao, A. (2002). Epistemic levels in argument: An analysis of university oceanography students’ use of evidence in writing. Science Education, 86, 314–342.

[ece36776-bib-0026] Kuh, G. D. (2008). High‐impact educational practices: What they are, who has access to them, and why they matter. Washington, DC: Association of American Colleges and Universities.

[ece36776-bib-0027] Lemons, P. P. , & Lemons, J. D. (2013). Questions for assessing higher‐order cognitive skills: It’s not just bloom’s. CBE Life Sciences Education, 12(1), 47–58. 10.1187/cbe.12-03-0024 23463228 PMC3587855

[ece36776-bib-0028] Lemons, P. P. , Reynolds, J. A. , Curtin, A. , & Bissell, A. (2013). Improving critical‐thinking skills in introductory biology through quality practice and metacognition In Kaplan M., Silver N., LaVaque‐Manty D., & Meizlish D. (Eds.), Using reflection and metacognition to improve student learning. Sterling, VA: Stylus Publishing.

[ece36776-bib-0029] Lerch, C. , Bilics, A. , & Colley, B. (2006). Using reflection to develop higher order processes. Paper presented at the Annual Meeting of the American Education Research Association, San Francisco, CA.

[ece36776-bib-0030] Lerner, N. (2007). Laboratory lessons for writing and science. Written Communication, 24, 191–222.

[ece36776-bib-0031] Li, L. , Liu, X. , & Steckelberg, A. L. (2010). Assessor or Assessee: How student learning improves by giving and receiving peer feedback. British Journal of Educational Technology, 41(3), 525–536. 10.1111/j.1467-8535.2009.00968.x

[ece36776-bib-0032] Loertscher, J. , Green, D. , Lewis, J. E. , Lin, S. , & Minderhout, V. (2014). Identification of threshold concepts for biochemistry. CBE Life Sciences Education, 13(3), 516–528. 10.1187/cbe.14-04-0066 25185234 PMC4152212

[ece36776-bib-0033] Lundstrom, K. , & Baker, W. (2009). To give is better than to receive: The benefits of peer review to the reviewer’s own writing. Journal of Second Language Writing, 18(1), 30–43. 10.1016/j.jslw.2008.06.002

[ece36776-bib-0034] Marion, S. B. , Schmid, L. , Carter, E. , Thompson, R. J. , Willis, J. , Mauger, L. , & Reynolds, J. A. (in prep). What Makes Biology Test Questions Challenging? Troublesome topics, cognitive skills, cognitive activities, question forms, and interaction effects.

[ece36776-bib-0035] Melzer, D. (2014). Assignments across the curriculum: A national study of college writing. Provo, UT: Utah State University Press.

[ece36776-bib-0036] Meyer, J. H. F. , & Land, R. E. (2005). Threshold concepts and troublesome knowledge (2): Epistemological considerations and a conceptual framework for teaching and learning. Higher Education, 49, 373–388. 10.1007/s10734-004-6779-5

[ece36776-bib-0037] Moon, A. , Gere, A. R. , & Shultz, G. V. (2018). Writing in the STEM Classroom: Faculty conceptions of writing and its role in the undergraduate classroom. Science Education, 102(5), 1007–1028. 10.1002/sce.21454

[ece36776-bib-0038] Reynolds, J. A. , & Moskovitz, C. (2008). Calibrated Peer Review™ assignments in science courses: Are they designed to promote critical thinking and writing skills? Journal of College Science Teaching, 38(2), 60–66.

[ece36776-bib-0039] Reynolds, J. A. , & Russell, V. (2008). Can you hear us now?: A comparison of peer review quality when students give audio versus written feedback. Writing Across the Curriculum Journal, 19, 29–44.

[ece36776-bib-0040] Reynolds, J. A. , Thaiss, C. , Katkin, W. , & Thompson, R. J., Jr. (2012). Writing to‐learn in undergraduate science education: A community‐based, conceptually‐driven approach. CBE – Life Science Education, 11(1), 17–25. 10.1187/cbe.11-08-0064 PMC3292059 22383613

[ece36776-bib-0041] Reynolds, J. A. , & Thompson, R. J. Jr (2011). Want to improve undergraduate thesis writing? Engage students and their faculty readers in scientific peer review. CBE ‐ Life Science Education, 10, 209–215. 10.1187/cbe.10-10-0127 PMC3105927 21633069

[ece36776-bib-0042] Ruegg, R. (2018). The effect of peer and teacher feedback on changes in EFL students’ writing self‐efficacy. The Language Learning Journal, 46(2), 87–102. 10.1080/09571736.2014.958190

[ece36776-bib-0043] Steele, C. M. (2011). Whistling Vivaldi: How stereotypes affect us and what we can do. New York, NY: W. W. Norton & Company.

[ece36776-bib-0044] Thompson, R. J. Jr. , Finkenstaedt‐Quinn, S. A. , Shultz, G. V. , Gere, A. R. , Schmid, L. , Dowd, J. E. , … Reynolds, J. E. (in review). How faculty discipline and beliefs influence instructional uses of writing in STEM undergraduate courses at research‐intensive universities. Journal of Writing Research.

[ece36776-bib-0045] Tsai, C. C. (2001). A review and discussion of epistemological commitments, metacognition, and critical thinking with suggestions on their enhancement in internet‐assisted chemistry classrooms. Journal of Chemical Education, 78(7), 970 10.1021/ed078p970

[ece36776-bib-0046] White, R. T. (1998). Decisions and problems in research on metacognition In Tobin K. G. (Ed.), International handbook of science education (pp. 1207–1213). Dordrecht, The Netherlands: Kluwer.

[ece36776-bib-0047] Williams, W. M. , Papierno, P. B. , Makel, M. C. , & Ceci, S. J. (2004). Thinking like a scientist about real‐world problems: The Cornell Institute for Research on Children Science Education Program. Journal of Applied Developmental Psychology, 25(1), 107–126. 10.1016/j.appdev.2003.11.002

